# Insights Into Adolescents' Substance Use in a Low–Middle-Income Country During the COVID-19 Pandemic

**DOI:** 10.3389/fpsyt.2021.739698

**Published:** 2021-10-14

**Authors:** Lee Thung Sen, Kristiana Siste, Enjeline Hanafi, Belinda Julivia Murtani, Hans Christian, Albert Prabowo Limawan, Adrian, Levina Putri Siswidiani

**Affiliations:** Department of Psychiatry, Faculty of Medicine, Universitas Indonesia—Dr. Cipto Mangunkusumo General Hospital, Jakarta, Indonesia

**Keywords:** adolescent, alcohol, cigarette, drugs, COVID-19, Indonesia

## Abstract

**Introduction:** The COVID-19 pandemic and its lockdown have been a significant life event for many individuals, particularly adolescents. The immense psychological pressure could drive risky behavior, e.g., substance use, while lockdown might lead to decreased use. This study aimed to observe the change in substance use among adolescents in Indonesia and the moderating variables to consumption during the COVID-19 lockdown period.

**Methods:** This study utilized an online survey from April 28, 2020 to June 30, 2020. The hyperlink was disseminated to school administrators and parenting groups through social media and direct messages. A total of 2,932 adolescents (17.4 ± 2.24 and 78.7% females) submitted valid responses. The survey was comprised of a sociodemographic section, substance use details, and psychometric sections, including the Alcohol Use Disorders Identification Test (AUDIT), Cigarette Dependence Scale 12 (CDS-12), Pittsburgh Sleep Quality Index (PSQI), and Strength and Difficulties Questionnaire (SDQ).

**Results:** Overall, adolescent alcohol use during the pandemic was 5.1%, cigarette smoking was 3.1%, and drug consumption was 0.4%. Over half (53.4%) of alcohol drinkers reported increased drinking, and 33.1% had harmful or dependence-like drinking behavior; in contrast, 44.4% of adolescent smokers disclosed reduced cigarette consumption. Around 37.8% of the drug users indicated increased use. During the pandemic, adolescent alcohol use was associated with higher education [adjusted odds ratio (AOR) = 2.67, 95% confidence interval (CI) 1.02–4.86, *p* = 0.04], higher AUDIT scores (AOR = 1.33, 95% CI 1.25–1.42, *p* < 0.001), and very low prosocial behavior (AOR = 2.46, 95% CI 1.52–3.88, *p* < 0.001). Cigarette smoking was correlated with male sex (AOR = 9.56, 95% CI 5.64–16.62, *p* < 0.001), age (AOR = 1.40, 95% CI 1.14–1.75, *p* < 0.001), and higher CDS score (AOR = 1.17, 95% CI 1.13–1.20, *p* < 0.001).

**Conclusions:** Rates of adolescent substance use were significant, with sizeable proportions reporting higher usage. This appeared to occur predominantly in specific demographics and those with a lower protective psychosocial attribute, i.e., prosocial behavior, during the lockdown. These findings should urge the strengthening of adolescent addiction care during and after the pandemic.

## Introduction

The World Health Organization (WHO) declared the severe acute respiratory syndrome coronavirus 2 (SARS-CoV-2) as a global pandemic on March 11, 2020. As a countermeasure against the pandemic, the Indonesian government implemented a large-scale social restriction (*pembatasan sosial berskala besar*/PSBB) in April 2020. During the large-scale social restriction, public places, including offices and schools, were closed along with a massive reduction of running public transports (1). Despite the effort in reducing further COVID-19 transmission, 4 months later, the drawbacks of this policy rose as several psychological impacts were discovered. According to the survey held by the Indonesian Psychiatrist Association from April to August 2020, around 64% adolescents suffered at least one psychological problem such as anxiety, depression, or posttraumatic complaints during the pandemic (2). A similar trend was found in a Spanish survey, which noted a 34.7% increment in psychopathological problems among adolescents after the pandemic lockdown (3). These mental health problems among adolescents might have emerged due to the implementation of online learning, which limits social interaction with their peers (4–7). Adolescence is a transitional phase of growth and development in which adolescents would consider the relationship with their peers as sources of inclusivity, trust, affection, and self-esteem (8). Thus, they would feel more comfortable sharing their feelings to their peers rather than their parents at home (9, 10). These psychopathological problems would eventually affect adolescent's productivity. Abrupt online learning was believed to a decrease in study motivation, daily activity neglection, and also a rise in drop-outs (11). This stressful event was worsened by the uncertain and ever-changing policies for academic activities, such as exams, graduation, and exchange programs (6). In addition, financial problems have become another stressful event, as the world economy was heavily hit by the pandemic. Some students lost their part-time jobs, while their families were also struggling with unstable income during the pandemic (6, 11, 12).

For some individuals, these burdens may lead to unfavorable coping behavior, such as substance abuse (13). This correlation has been observed with the 2003 SARS outbreak, in which alcohol abuse/dependence symptoms were induced 3 years after being exposed to the outbreak. Unfortunately, the population in this study were hospital employees aged 33–35 years old, and there has been no research accounting for the adolescent population (14).

Before the COVID-19 pandemic, in 2016, among Indonesians older than 15 years old, the prevalence of heavy episodic drinking (pure alcohol consumption of at least 60 g on at least one occasion during the past 30 days) was 6.5%, with the overall prevalence of alcohol use disorders at 0.8% and alcohol dependence at 0.7% (15). Meanwhile, for tobacco smoking, the Indonesian 2018 Basic Health Research stated that the prevalence of tobacco use among Indonesians older than 15 years old was 33.8%, with daily tobacco smoking at 24.3% and e-cigarette use at about 2.8% (16). As for psychoactive drugs, according to the Indonesian Drugs Report 2019, the prevalence of drug abuse among students in 2018 was 3.2%, ~2 million individuals (17). The current study explored the impact of physical distancing toward psychoactive substance usage, including their related factors, as a response to the concerning number of substance abuse among Indonesian adolescents and the possible emergence of new substitutes, such as new or homemade substances. This study's results would improve our understanding of the management of substance abuse in this “new normal” era. Changes in substance use behavior during the pandemic could be unpredictable, as emotional distress, isolation, and unemployment drove the demand for substance use as a coping mechanism, while reduced availability, escalating prices, and financial limitations decreased substance usage (18).

## Methods

### Respondents

School administrators, teachers, and parents were approached, as contact points, through direct correspondences, emails, and social media [e.g., instant messaging applications (WhatsApp or Line)] and the research link was shared. Upon guardian or parental consent, the contact points continued the link to the respondents. The first page of the survey explained the purpose and mechanics of the study, including management of privacy and data, and requested written assent [in line with respecting subjects' autonomy (19) and would be omitted from the study should they reject to participate]. The contact points of each school and parents were urged to pass on the survey link to other parents and teachers. Inclusion criteria for respondents were (i) provided emails (names were not requested) to prevent multiple responses, (ii) aged 10–20 years old, (iii) currently residing in Indonesia, and (iv) capable of understanding Bahasa Indonesia. The selected age range for adolescents in this study was adapted from the WHO definition of 10–19 years old (20) and the Indonesian Pediatric Association of 10–20 years old (21). This study defined early adolescence as 10–14 years old, mid-adolescence as 15–17 years old, and late adolescence as 18–20 years old. Several responses of non-consenting (*n* = 30), duplicates (*n* = 23), and non-Indonesia residents (*n* = 10) were removed. The survey was part of a larger study targeting both adults and adolescents, which separated psychopathology measures between the Symptoms Checklist 90 (for adults) and the Strength and Difficulties Questionnaire (SDQ; for adolescents). However, around 40 respondents mistakenly answered the Symptoms Checklist 90 (SCL-90) and were removed from all analyses. Personal information (e.g., emails) was only accessible to the researcher; they were only inspected for duplicates and deleted prior to further data examination. Overall, a total of 2,932 respondents completed the survey, representing 33 of 34 provinces in Indonesia and all seven main islands (Java 78.5%, Sumatera 8.3%, Kalimantan 0.6%, Sulawesi 9.7%, Nusa Tenggara and Bali 2.6%, Papua 0.1%, and Maluku 0.2%) across Indonesia.

### Procedures

The authors designed an online survey employing *Google Form*. A shortened hyperlink was generated and publicized by the research team through social media and direct correspondences to several schools across Indonesia and parenting groups between April 28, 2020 and June 30, 2020. Upon clicking the survey link, the survey started with a title page containing an outline of the study's purpose, respondents' inclusion criteria, and data management. Teachers, guardians, and parents were advised to read through the study's description before allowing their children/students to answer the survey. Each respondent was asked for written informed consent, and an author's email for correspondence was provided for further information and should respondents wish for subsequent clinical assessment/therapy. Those who did not give consent were directed to finish without filling the survey. The survey contained a sociodemographic section (gender, age, monthly household income, education level, occupations, province of residence, and the number of adults currently residing with the participant), followed by quarantine-related questions (the practice of quarantine and physical distancing, location of quarantine, living companion during quarantine, and confirmed/suspected cases within the household) and substance use consumption detail [alcohol, daily cigarette, and drug consumptions since the start of COVID-19 pandemic in Indonesia (March 2, 2020). The option “did not consume” was described as not consuming any substances at all since the beginning of the pandemic, while “consume” was described as having consumed any amount of substances since the beginning of the pandemic. For those who answered having consumed any of the three substances, their perceived change (unchanged, increased, or decreased) of current use compared to before the pandemic was captured]. In the last section, respondents who consumed alcohol were asked to complete the Alcohol Use Disorders Identification Test (AUDIT) and Cigarette Dependence Scale (CDS) for those who consumed cigarettes. There was yet no validated self-report instrument for measuring drug use severity in Indonesia. All respondents were required to complete the Pittsburgh Sleep Quality Index (PSQI) and SDQ. The survey was separated into several sections and span around 14 web pages (since several instruments were divided into multiple sections) and required about 40–50 min for completion. However, response duration could not be evaluated in *Google Form* to prevent reporting bias. All items were marked mandatory; thus, respondents could not continue to the next section or submit the survey if there was an unanswered item.

Physical distancing as an extension of self-quarantine included several practices defined in this study as studying/working from home, alternate studying/working days, and/or other physical distancing practices as per the guideline from the Indonesian COVID-19 Response Acceleration Task Force (GTPP COVID-19). Respondents were asked whether themselves and/or any household member had been declared as COVID-19 suspect cases and/or diagnosed with COVID-19, following the descriptions provided by the GTPP COVID-19, Indonesian Ministry of Health, and World Health Organization. Province of residence was categorized into whether PSBB had been implemented at the commencement of the study (April 28, 2020) based on data from GTPP COVID-19, which included DKI Jakarta, West Java, East Java, Central Java, Banten, West Kalimantan, North Kalimantan, Gorontalo, West Sumatera, Riau, and South Sulawesi. Income levels were divided based on classification by the World Bank.

### Psychometric Tools

#### Alcohol Use Disorders Identification Test

This questionnaire was developed as a screening instrument to identify the effects of dependence and harmful alcohol use, designed to be used in primary health care and applicable for international use. This questionnaire comprises 10 questions focusing on the recent use of alcohol; scoring ranges from 0 to 40 with a score of 8–14 interpreted as harmful alcohol use and ≥15 as a possibility for dependence (22). The WHO collaborative study showed that AUDIT is a valid instrument in six countries with a sensitivity of 92% and a specificity of 94% (23). AUDIT had been validated among adolescents (24, 25), with a suggested threshold of 2 for detecting problematic use and 3 for the likelihood of any disorder (25). The Cronbach's alpha in this study was 0.86, among 148 respondents consuming alcohol. The Indonesian version of AUDIT has a Cronbach's alpha = 0.859 (26).

#### Cigarette Dependence Scale 12

CDS is a self-reported questionnaire that aids in determining the severity of nicotine dependence (27). Each question has five multiple-choice answers. Question number 1 asked cigarette dependency, scoring 0 to 100 and divided into five intervals (0–20, 21–40, 41–60, 61–80, and 81–100). Question number 2 asked the number of cigarettes smoked, ranging from 0 to more than 30 rolls divided into succeeding five intervals (e.g., 0–5 and 6–10). Question number 3 asked about how soon after waking up the respondents smoke his or her first cigarette. This question used a Likert scale with values from 1 to 5, from “very easy” to “impossible.” Meanwhile, the Likert scale used in the rest of the questions was from “completely disagree” to “highly agree.” The output of this questionnaire is in a numeric form with no determined cutoff number, and a higher score indicates more severe nicotine dependence. Evaluation of the Indonesian version of CDS showed that a modification of the CDS from 12 to 10 (items 3 and 9 were removed) improved the instrument's statistical value with good reliability, Cronbach's alpha = 0.91, and intraclass correlation coefficient = 0.91 (28). The CDS was comparably validated within a population of teenage smokers (27). The reliability in this study was 0.91 among 90 smoking respondents.

#### Pittsburgh Sleep Quality Index

The PSQI is a commonly used instrument to assess sleep quality in clinical or non-clinical subjects and adolescents with good internal reliabilities of α = 0.73–0.85 (29, 30). The questionnaire consists of 24 items, divided into 20 multiple choices and four open-ended questions. About five of 24 items need assessment from a partner or another individual on the subject's sleep pattern. Another 19 items were self-answered questions and can be grouped into seven components, with each being measured between 0 and 3 (maximum 21). A score >5 indicates poor sleep quality. The Indonesian version of the PSQI has been validated with a reliability of α = 0.79, content validity of 0.89, and specificity of 81% (31). The Cronbach's alpha in this study was 0.77.

#### Strength and Difficulties Questionnaire

The SDQ is a questionnaire for children and youths (32–34). The questionnaire consists of 25 items regarding children's behavior in the past 6 months. Those items are divided into five subscales: hyperactivity, emotional symptoms, conduct problems, peer problems, and prosocial behaviors. Each item was marked with “Not True” (=0), “Somewhat True” (=1), and “Certainly True” (=2). Scores of “Not True” and “Somewhat True” are reversed for the prosocial behavior subscale. The total score for each subscale is generated by summing the scores for the five items, thereby resulting in a score ranging from 0 to 10 (32). SDQ scores are divided into four bands, namely, 80% “close to average,” 10% “slightly raised/lowered,” 5% “high/low,” and 5% “very high/very low.” The Indonesian version of the SDQ has a sensitivity of 67% and a specificity of 68%, with α = 0.77 (33). The reliability in this study was α = 0.75.

### Data Analysis

Data was analyzed using SPSS version 27.0 (IBM, USA) and R Essentials Statistics for SPSS 27.0 utilizing R version 3.6.3. A descriptive analysis was performed for all data. Categorical data was compared using chi-square and *z*-test column proportions utilizing Bonferroni correction for multiple pair comparisons. Univariate and multivariate logistic regressions were performed for sociodemographic factors, quarantine and COVID-19-related elements, and psychometric results. Firth's penalized maximum likelihood regression was utilized to overcome the small-event bias for both alcohol and cigarette consumptions (35). Alcohol and cigarette consumptions were categorized into binary (consuming/not consuming) for regression analysis. Reference category was not consuming alcohol or cigarette during the COVID-19 pandemic. Drug consumption had very small frequencies even after dichotomization and was refrained from similar scrutiny. Results were deemed significant if *p* < 0.05 and scrutinizing the 95% confidence interval (CI).

### Ethical Approval

This study was approved by the Institutional Ethics Committee of the Faculty of Medicine, Universitas Indonesia—Dr. Cipto Mangunkusumo General Hospital (KET-413/UN2.F1/ETIK/PPM/00/02/2020). Digital written consents were acquired from all responses.

## Results

### Sociodemographic and Usage Prevalence

Overall, of the 2,932 respondents, 21.3% were male and the mean age was 17.4 ± 2.24. Around 30.5% attained up to junior high school and 7.1% had reached higher education. The majority, 56.5%, of respondents were non-university students, 84.9% lived in provinces implementing PSBB, and 96.1% practiced physical distancing measures. Around 3.5% (*N* = 103) of respondents reported having positive or suspected cases within their household.

The prevalence of alcohol drinking among Indonesian adolescents during the COVID-19 pandemic and quarantine period was 5.1%, 3.1% for cigarette usage, and 0.4% for drug consumption. The mean age of alcohol drinkers was 17.6 ± 2.30, while among smokers was 18.1 ± 1.76. Of those who consumed alcohol, 25.7% reported unchanged consumption, 53.4% increased drinking, and 20.9% decreased usage. Among the smoking respondents, 37.8% disclosed unchanged cigarette consumption, 17.8% increased smoking, and 44.4% decreased usage. Among those who disclosed drug consumption, 53.8% reported unchanged consumption, 30.8% increased drug use, and 15.4% decreased drug use.

### Descriptive Psychometric

This study found that 53.4% of alcohol using respondents perceived heightened alcohol use during the COVID-19 pandemic (see Table 1). More late adolescents were found among those with increased alcohol consumption group than the unchanged alcohol consumption group (74.7 and 65.8%, respectively). The greater proportion of respondents with increased alcohol use originated from low-income households (50.6%) compared to the alcohol unchanged group (31.6%).

**Table 1:** Descriptive data stratified by alcohol consumption.

**Variables**		**Did not consume (*N* = 2,784)**	**Alcohol consumption change**						***X*** ^**2**^
			**Unchanged^a^**		**Increased^b^**		**Decreased^c^**		
			**(*****N*** **= 38)**		**(*****N*** **= 79)**		**(*****N*** **= 31)**		
		***n***	***n***	**%**	***n***	**%**	***n***	**%**	
**Sex**									
	Male	578	11	28.9	21	26.6	15	48.4	5.07
	Female	2,206	27	71.1	58	73.4	16	51.6	
**Age**									
	Early adolescent	343	8	21.1	9	11.4	3	9.7	3.05
	Mid adolescent	619	5	13.2	11	13.9	3	9.7	
	Late adolescent	1,822	25	65.8	59	74.7	25	80.6	
**Education**									
	Up to junior high	858	12	31.6	18	22.8	7	22.6	1.45
	High school	1,734	23	60.5	52	65.8	20	64.5	
	Higher studies	192	3	7.9	9	11.4	4	12.9	
**Occupation**									
	Non-university students	1,580	24	63.2	48	60.8	12	38.7	11.24
	University students	1,107	12	31.6	24	30.4	17	54.8	
	Employed	96	2	5.3	7	8.9	1	3.2	
	NEET^d^	1	0	0.0	0	0.0	1	3.2	
**Household monthly income**									
	Low	989	12	31.6	40	50.6	8	25.8	21.09^**^
	Lower middle	1,242	11	28.9	31	39.2	13	41.9	
	Upper Middle	417	6	15.8	7	8.9	4	12.9	
	High	136	9	23.7	1	1.3^a,c^	6	19.4	
**Adults within household**									
	0	33	1	2.6	3	3.8	2	6.5	0.67
	1–2	936	14	36.8	26	32.9	8	25.8	
	3–5	1,562	20	52.6	43	54.4	18	58.1	
	>5	253	3	7.9	7	8.9	3	9.7	
**Region**									
	Implemented PSBB^e^	2,368	34	89.5	68	86.1	20	64.5	5.97
	Has not implemented PSBB^e^	416	4	10.5	11	13.9	11	35.5	
**Physical distancing**									
	Practiced	2,678	36	94.7	75	94.9	30	96.8	0.2
	Did not practice	106	2	5.3	4	5.1	1	3.2	
**Positive/suspect case in household**									
	Yes	98	2	5.3	2	2.5	1	3.2	0.59
	No	2,686	36	94.7	77	97.5	30	96.8	
**AUDIT cat**									
	Normal		22	57.9^b,c^	68	86.1^a,c^	9	29.0^a,b^	35.53^***^
	Harmful alcohol use		3	7.9	3	3.8	3	9.7	
	Likelihood of any alcohol disorder		13	34.2	8	10.1^a,c^	19	61.3	
**PSQI**									
	Normal	1,517	20	52.6	53	67.1	14	45.2	5.22
	Poor sleep quality	1,267	18	47.4	26	32.9	17	54.8	
**Emotional problems**									
	Close to average	1,583	21	55.3	50	63.3	18	58.1	8.81
	Slightly raised	370	1	2.6	9	11.4	2	6.5	
	High	260	4	10.5	3	3.8	5	16.1	
	Very high	571	12	31.6	17	21.5	6	19.4	
**Conduct problems**									
	Close to average	2,510	34	89.5	69	87.3	25	80.6	5.87
	Slightly raised	168	0	0.0	3	3.8	3	9.7	
	High	45	1	2.6	2	2.5	2	6.5	
	Very high	61	3	7.9	5	6.3	1	3.2	
**Hyperactivity**									
	Close to average	2,124	33	86.8	62	78.5	21	67.7	16.82^**^
	Slightly raised	359	3	0.8	12	15.2	6	19.4^a,b^	
	High	199	2	1.0	5	6.3	3	9.7	
	Very high	102	0	0.0	0	0.0	1	3.2	
**Peer problems**									
	Close to average	2,310	31	81.6	62	78.5	26	83.9	8.63
	Slightly raised	254	1	2.6	11	13.9	1	3.2	
	High	162	5	13.2	4	5.1	2	6.5	
	Very high	58	1	2.6	2	2.5	2	6.5	
**Prosocial behaviors**									
	Close to average	243	7	18.4	16	20.3	6	19.4	2.46
	Slightly decreased	166	4	10.5	5	6.3	1	3.2	
	Low	262	6	15.8	8	10.1	4	12.9	
	Very low	2,113	21	55.3	50	63.3	20	64.5	

a,b,c *Significant difference Bonferroni corrected;*

d *not in employment, education, or training;*

e *large-scale social distancing;*

** *p ≤ 0.01;*

*** *p ≤ 0.001*.

The proportion of respondents showing very high emotional symptoms were lower in the increased alcohol consumption subgroup than in the unchanged alcohol consumption subgroup (21.5 and 31.6%, respectively). Moreover, the proportion of respondents possessing very low prosocial behaviors were lower in the increased alcohol use subgroup compared to the stable alcohol drinking subgroup (18.4 and 20.3%, respectively).

Across all drinking fluctuations, the AUDIT scores demonstrated that 6.1% (2.8 ± 0.15) had harmful drinking and 27.0% (9.4 ± 0.97) had a likelihood of any alcohol disorder. Based on the AUDIT scores, the proportion of respondents drinking problematically in the stable drinking subgroup [7.9% (3 ± 0)] was higher than that in the increased alcohol consumption subgroup [3.8% (2.7 ± 0.33)]. A greater proportion of respondents having a likelihood to be disordered was also found in the unchanged alcohol consumption group rather than in the increased alcohol consumption group [34.2% (6.9 ± 0.99) and 10.1% (13.1 ± 3.31), respectively]. In addition, sleep problems and emotional problems were also found in both the unchanged alcohol consumption group and the increased alcohol consumption group. Overall, PSQI and emotional symptoms score for respondents who drank alcohol was 5.47 ± 3.04 and 4.04 ± 3.07, respectively (Table 2).

**Table 2:** Descriptive scores of all psychometric tests across different respondent groups.

**Variables**	**Alcohol drinkers**	**Cigarette smokers**	**Drugs consumers**
1. AUDIT	2.72 ± 5.21	–	–
2. CDS	–	20.03 ± 8.57	–
3. PSQI	5.47 ± 3.04	6.07 ± 3.54	9.69 ± 3.33
4. Emotional problems	4.04 ± 3.07	4.00 ± 3.04	6.92 ± 2.87
5. Conduct problems	2.80 ± 1.88	2.97 ± 1.97	3.00 ± 2.04
6. Hyperactivity problems	3.67 ± 1.76	3.72 ± 1.81	5.54 ± 1.85
7. Peer problems	3.34 ± 1.64	3.53 ± 1.80	3.15 ± 1.52
8. Prosocial behaviors	6.74 ± 3.07	6.93 ± 2.97	7.77 ± 1.79

*Data presented as mean ± SD*.

Table 3 depicts the descriptive distribution of adolescent smokers. Most of the smokers were male (68.8%) and in their late adolescents (82.2%). More adolescents reported to have less sleep disturbance (37.5%), less emotional symptoms (18.8%), and greater score on prosocial behavior (25.0%) among the increased cigarette consumption group compared to the decreased cigarette consumption group, with around 60.0% reporting a decline in sleep quality, 27.5% had very high scores of emotional symptoms, and 15.0% very low scores on prosocial behavior. CDS score differed significantly between the three groups of smoking consumption changes [*F*_(2, 87)_ = 4.53, *p* = 0.013]. The mean CDS score among smokers with unchanged consumption was 16.7 ± 5.75, increased smoking 22.3 ± 10.58, and decreased smoking 22.0 ± 8.98. A *post-hoc* analysis demonstrated a significant difference between decreased and unchanged smoking (*p* = 0.008); *post-hoc* analyses for other combinations did not yield significant results.

**Table 3:** Descriptive data stratified by cigarette consumption.

**Variables**		**Cigarette consumption change**							***X*** ^**2**^
		**Did not consume**	**Unchanged** ***^a^***		**Increased^b^**		**Decreased** ***^c^***		
		**(*N* = 2,833)**	**(*****N*** **= 34)**		**(*****N*** **= 16)**		**(*****N*** **= 40)**		
		***n***	***n***	**%**	***n***	**%**	***n***	**%**	
**Sex**									
	Male	563	20	58.8	10	62.5	32	80.0	4.22
	Female	2,279	14	41.2	6	37.5	8	20.0	
**Age**									
	Early adolescent	360	1	2.9	0	0.0	2	5.0	1.33
	Mid adolescent	625	4	11.8	3	18.8	6	15.0	
	Late adolescent	1,857	29	85.3	13	81.3	32	80.0	
**Education**									
	Up to junior high	880	5	14.7	2	12.5	8	20.0	2.16
	High school	1,765	23	67.6	12	75.0	29	72.5	
	Higher studies	197	6	17.6	2	12.5	3	7.5	
**Occupation**									
	Non-university students	1,621	14	41.2	6	37.5	23	57.5	5.09
	University students	1,122	18	52.9	7	43.8	13	32.5	
	Employed	97	2	5.9	3	18.8	4	10.0	
	NEET^d^	2	0	0.0	0	0.0	0	0.0	
**Household monthly income**									
	Low	1,018	15	44.1	4	25.0	12	30.0	8.54
	Lower middle	1,258	13	38.2	10	62.5	16	40.0	
	Upper middle	420	5	14.7	0	0.0	9	22.5	
	High	146	1	2.9	2	12.5	3	7.5	
**Adults within household**									
	0	37	1	2.9	1	6.3	0	0.0	4.18
	1–2	958	8	23.5	6	37.5	12	30.0	
	3–5	1,587	23	67.6	8	50.0	25	62.5	
	>5	260	2	5.9	1	6.3	3	7.5	
**Region**									
	Implemented PSBB^e^	2,421	25	73.5	11	68.8	33	82.5^a,b^	17.60^***^
	Has not implemented PSBB^e^	421	9	26.5	5	31.3	7	17.5^a,b^	
**Physical distancing**									
	Practiced	2,732	33	97.1	16	100.0	38	95.0	0.91
	Did not practice	110	1	2.9	0	0	2	5.0	
**Positive/suspect case in household**									
	Yes	98	2	5.9	2	12.5	1	2.5	2.19
	No	2,744	32	94.1	14	87.5	39	97.5	
**PSQI**									
	Normal	1,557	21	61.8	10	62.5	16	40.0	4.31
	Poor sleep quality	1,285	13	38.2	6	37.5	24	60.0	
**Emotional problems**									
	Close to average	1,618	20	58.8	13	81.3	21	52.5	6.26
	Slightly raised	376	2	5.9	0	0.0	4	10.0	
	High	263	5	14.7	0	0.0	4	10.0	
	Very high	585	7	20.6	3	18.8	11	27.5	
**Conduct problems**									
	Close to average	2,564	30	96.8	11	100.0	33	94.3	5.85
	Slightly raised	173	0	0.0	0	0.0	1	2.9	
	High	49	1	3.2	0	0.0	0	0.0	
	Very high	69	0	0.0	0	0.0	1	2.9	
**Hyperactivity**									
	Close to average	2,162	30	88.2	14	87.5	34	85.0	2.87
	Slightly raised	370	4	11.8	2	12.5	4	10.0	
	High	207	0	0.0	0	0.0	2	5.0	
	Very high	103	0	0.0	0	0.0	0	0.0	
**Peer problems**									
	Close to average	2,359	29	85.3	13	81.3	28	70.0	9.45
	Slightly raised	260	2	5.9	1	6.3	4	10.0	
	High	166	3	8.8	2	12.5	2	5.0	
	Very high	57	0	0.0	0	0.0	6	15.0	
**Prosocial behaviors**									
	Close to average	254	8	23.5	4	25.0	6	15.0	6.21
	Slightly decreased	167	3	8.8	1	6.3	5	12.5	
	Low	271	6	17.6	0	0.0	3	7.5	
	Very low	2,150	17	50.0	11	68.8	26	65.0	

a,b,c *Significant difference Bonferroni corrected;*

d *not in employment, education, or training;*

e *large-scale social distancing;*

*** *p ≤ 0.001*.

Among those who consumed drugs, about 30.8% (*N* = 4) consumed at least two types of drugs. Overall, 11.1% used cannabis, 11.1% sedatives or inhalants, 5.6% cocaine, 11.1% other stimulants (e.g., amphetamines), and 61.1% other drugs (e.g., opiates, steroid, and abused prescription). About 84.6% of respondents who reported consuming drugs were female and 76.9% were late adolescents, but were not statistically significant comparing in-between subgroups. About three-fourths (75.0%) of those disclosing increased drug consumption reside in non-PSBB provinces, and all respondents with decreased drug usage were living in PSBB provinces, and similar proportions were reported for sleeping problems in both groups. Half of those reporting increased drug use scored very highly on emotional symptoms, while 71.4% of those having unchanged consumptions also scored very highly on emotional symptoms and 28.6% on hyperactivity trait (Table 4). Overall emotional symptoms score was 6.92 ± 2.87 and hyperactivity was 5.54 ± 1.85. Respondents reporting consuming drugs scored the highest on PSQI, 9.69 ± 3.33 (Table 2).

**Table 4:** Descriptive data stratified by drug use.

**Variables**		**Drugs consumption change**							***X*** ^**2**^
		**Did not consume**	**Unchanged^a^**		**Increased^b^**		**Decreased^c^**		
		**(*N* = 2,919)**	**(*****N*** **= 7)**		**(*****N*** **= 4)**		**(*****N*** **= 2)**		
		***n***	***n***	**%**	***n***	**%**	***n***	**%**	
**Sex**									
	Male	623	0	0.0	1	25.0	1	50.0	3.4
	Female	2,296	7	100.0	3	75.0	1	50.0	
**Age**									
	Early adolescent	361	2	28.6	0	0.0	0	0.0	4.13
	Mid adolescent	637	0	0.0	1	25.0	0	0.0	
	Late adolescent	1,921	5	71.4	3	75.0	2	100.0	
**Education**									
	Up to junior high	892	2	28.6	1	25.0	0	0.0	0.73
	High school	1,819	5	71.4	3	75.0	2	100.0	
	Higher studies	208	0	0.0	0	0.0	0	0.0	
**Occupation**									
	Non-university students	1,659	2	28.6	2	50.0	1	50.0	0.63
	University students	1,152	5	71.4	2	50.0	1	50.0	
	Employed	106	0	0.0	0	0.0	0	0.0	
	NEET^d^	2	0	0.0	0	0.0	0	0.0	
**Household monthly income**									
	Low	1,046	2	28.6	1	25.0	0	0.0	3.14
	Lower middle	1,292	2	28.6	2	50.0	1	50.0	
	Upper middle	432	1	14.3	1	25.0	0	0.0	
	High	149	2	28.6	0	0.0	1	50.0	
**Adults within household**									
	0	39	0	0.0	0	0.0	0	0.0	0
	1–2	982	1	14.3	1	25.0	0	0.0	
	3–5	1,634	6	85.7	2	50.0	1	50.0	
	>5	264	0	0.0	1	25.0	1	50.0	
**Region**									
	Implemented PSBB^e^	2,484	3	42.9	1	25.0	2	100.0	1.13
	Has not implemented PSBB^e^	435	4	57.1	3	75.0	0	0.0	
**Physical distancing**									
	Practiced	2,806	7	100.0	4	100.0	2	100.0	0
	Did not practice	113	0	0.0	0	0.0	0	0.0	
**Positive/suspect case in household**									
	Yes	75	1	14.3	0	0.0	0	0.0	0.65
	No	2,844	6	85.7	4	100.0	2	100.0	
**PSQI**									
	Normal	1,602	1	14.3	1	25.0	0	0.0	0.65
	Poor sleep quality	1,317	6	85.7	3	75.0	2	100.0	
**Emotional problems**									
	Close to average	1,670	1	14.3	0	0.0	1	50.0	7.10
	Slightly raised	380	0	0.0	1	25.0	1	50.0	
	High	270	1	14.3	1	25.0	0	0.0	
	Very high	599	5	71.4	2	50.0	0	0.0	
**Conduct problems**									
	Close to average	2,627	7	100.0	3	75.0	1	50.0	8.27
	Slightly raised	173	0	0	1	25.0	0	0.0	
	High	50	0	0	0	0	0	0.0	
	Very high	69	0	0	0	0	1	50.0	
**Hyperactivity**									
	Close to average	2,238	1	14.3	1	25.0	0	0.0	3.14
	Slightly raised	375	2	28.6	2	50.0	1	50.0	
	High	206	2	28.6	1	25.0	0	0.0	
	Very high	100	2	28.6	0	0.0	1	50.0	
**Peer problems**									
	Close to average	2,418	7	100.0	3	75.0	1	50.0	8.27
	Slightly raised	266	0	0	1	25.0	0	0.0	
	High	172	0	0	0	0.0	1	50.0	
	Very high	63	0	0	0	0.0	0	0.0	
**Prosocial behaviors**									
	Close to average	272	0	0.0	0	0.0	0	0.0	1.60
	Slightly decreased	174	1	14.3	1	25.0	0	0.0	
	Low	278	1	14.3	1	25.0	0	0.0	
	Very low	2,195	5	71.4	2	50.0	2	100.0	

a,b,c *Significant difference Bonferroni corrected;*

d *not in employment, education, or training;*

e *large-scale social distancing*.

### Correlates of Substance Consumption

As depicted in Table 5, alcohol consumption during the pandemic was correlated to higher-studies education level [adjusted odds ratio (AOR) = 2.67, 95% CI 1.02–4.86, *p* = 0.04], occupational status [not in education, employment, or training (NEET), AOR = 22.10, 95% CI 1.66–295.37, *p* = 0.02], higher AUDIT scores (AOR = 1.33, 95% CI 1.25–1.42, *p* < 0.001), and slightly decreased (AOR = 2.09, 95% CI 1.19–3.50, *p* = 0.01) and very low prosocial behavior (AOR = 2.46, 95% CI 1.52–3.88, *p* < 0.001), compared to non-alcohol consumption. In regard to cigarette, smoking during pandemic was associated with the male sex (AOR = 9.56, 95% CI 5.64–16.62, *p* < 0.001), increasing age (AOR = 1.40, 95% CI 1.14–1.75, *p* < 0.001), and higher CDS score (AOR = 1.17, 95% CI 1.13–1.20, *p* < 0.001).

**Table 5:** Regression analysis on alcohol and cigarette consumptions.

**Variables**	**Alcohol consumption**		**Cigarette consumption**	
	**cOR^a^**	**aOR^b^**	**cOR^a^**	**aOR^b^**
Sex (ref: female)	1.78 (1.24–2.54)^**^	1.74 (1.17–2.55)^***^	8.96 (5.68–14.14)^***^	13.81 (8.46–23.11)^***^
Age	1.04 (0.96–1.12)	1.05 (0.92–1.20)	1.19 (1.07–1.33)^***^	1.44 (1.19–1.75)^***^
**Education (ref: up to junior high)**				
High school	1.27 (0.86–1.87)	1.39 (0.80–2.40)	2.13 (1.21–3.75)^**^	1.40 (0.65–3.06)
Higher studies	1.93 (1.05–3.55)^*^	1.87 (0.86–3.94)	3.28 (1.48–7.24)^**^	2.07 (0.72–5.72)
**Occupation (ref: non-university students)**				
Employed	1.96 (0.99–3.90)	1.38 (0.63–2.82)	3.50 (1.66–7.38)^***^	1.50 (0.57–3.67)
NEET^c^	18.81 (1.17–303.34)^*^	17.37 (1.32–229.43)^*^	–	4.77 (0.03–72.49)
**Adults within household (ref = ≥1)**				
0	3.52 (1.45–8.54)^**^	3.34 (1.26–7.66)^*^	1.72 (0.41–7.26)	1.01 (0.17–3.84)
**Household monthly income (ref: low)**				
High	1.94 (1.09–3.46)^*^	2.21 (1.19–3.91)^**^	1.35 (0.55–3.29)	1.07 (0.38–2.61)
**Conduct problems (ref: close to average)**				
Very high	2.89 (1.41–5.96)^**^	2.84 (1.07–6.99)^*^	3.85 (1.71–8.69)^***^	3.10 (0.99–9.06)
**Hyperactivity (ref: close to average)**				
High	1.57 (0.90–0.275)	1.25 (0.62–2.38)	2.81 (1.57–5.04)^***^	2.67 (1.23–5.54)^**^
**Peer problems (ref: close to average)**				
Very high	1.67 (0.66–4.25)	1.16 (0.39–2.83)	3.55 (1.48–8.50)^**^	2.65 (0.90–6.90)
**Prosocial behaviors (ref: close to average)**				
Very low	0.36 (0.23–0.56)^***^	0.41 (0.26–0.66)^***^	0.35 (0.21–0.61)^***^	0.53 (0.29–1.00)

a *Crude odds ratio;*

b *adjusted odds ratio;*

c *not in employment, education, or training;*

* *p < 0.05;*

** *p < 0.01;*

*** *p < 0.001*.

## Discussion

In general, substance use among adolescents in Indonesia during the COVID-19 pandemic showed mixed fluctuations. Although some decreased their usage, a considerable proportion increased or maintained their consumption. The rate of substance use differed for each type of substance, with the highest figure being alcohol use, followed by cigarettes and, lastly, drug consumption. Naturally, adolescence is a transitional phase of autonomy confirmation, peer relevance, and experimentation on life choices (8), which, combined with the financial and social perturbations (6, 11) during the pandemic, might predispose them to greater risks. Additionally, brain development still occurs during the adolescence period; thus, teenagers tend to act impulsively without reflective thinking and more vulnerable to addictive behaviors. Adolescent brain is also sensitive to the effect of psychoactive substances; therefore, it may damage the nervous system and affect brain functioning (36, 37). These composite heightened vulnerabilities were reflected as higher substance use in a past national adolescent survey (17) and resonated in this study. Certain variables, AUDIT and CDS scores, education level, and low prosocial tendencies, were associated with either alcohol or cigarette consumption during the COVID-19 pandemic.

### Alcohol

The alcohol consumption among the current sample of adolescents seemed to demonstrate an increase compared to rates before the pandemic. This pattern was also noted to be linked with the male sex, the number of household adults, monthly income band, and scores of conduct problems and prosocial behavior. Past figures prior to the pandemic described a rate of 2.5% (38) for past-month alcohol drinking among Indonesian adolescents, half of the currently detected figure of 5.1%. Although the duration range utilized might also account for the difference, the rate of lifetime drinking was similarly small at 2.2% (38). Another global study among Indonesian school students noted a prevalence of 4.4% on current alcohol use (39), suggesting a potential increase in alcohol consumption during the pandemic. There was, however, a scarcity of data on alcohol abuse or dependence specifically among Indonesian adolescents. In comparison, the number of Canadian adolescents who consumed alcohol did not change significantly pre-and during the COVID-19 era; however, among those who drank alcohol, the frequency of alcohol use increased significantly (40). This resonated with the findings in this study, which elaborated that over half of the respondents reported increased alcohol consumption, 2-fold than those reporting decreased consumption.

The current study discovered a significant relationship between the male sex and alcohol consumption during the COVID-19 pandemic. Other studies during the COVID-19 pandemic noted similar findings (40, 41). Notably, adolescents in higher studies were more prone to consuming alcohol during the pandemic. Some recent data on college students suggested they were capable of sourcing hedonic stimulus from solitary use of substances (40, 42), which deviated from the past understanding of the peer contexts of adolescent substance use (43). This would resonate with findings of solitary drinking among adolescents during COVID-19 and speak volumes on the necessity to scrutinize further the source and procurement of substances among underage drinkers (with the legal age of alcohol purchase in Indonesia being 21). The oversight of alcohol sales could be considered loose in some low- and middle-income Asian countries, with a prior study illustrating that at least a third of minors being able to physically purchase alcoholic products (38), which should spur the scrutiny to digital alcohol sales. Astonishingly, there was also a finding on the use of virtual platform among peers for use of substances during COVID-19 (40).

The present study did not observe any correlation between household health status (proximity to COVID-19), the practice of physical distancing, and living in lockdown provinces. The maintenance of alcohol consumption during the pandemic was correlated to higher AUDIT scores, underscoring the vulnerabilities of those with an inclination of dependence, particularly as AUDIT has the predictive capacity of forming and sustaining problematic alcohol use among adolescents (24). Another study, albeit among adults, showed that low social connectedness and depressive symptoms were linked to increased past-month drinking during the pandemic (44), echoing the results of a meta-analysis on adolescents' coping motives and alcohol consumption (45). During the COVID-19 pandemic, individuals experienced decreased emotion regulation and hedonic tone, which could become the predictor factors of depressive symptoms. A common neurobiological pathway is also shared by both affective states and addictive disorder; thus, it may increase the risk for addictive behavior when an individual experiences mood disorders (46, 47). These findings were in line with the present study results, which noted that decreased and very low prosocial behaviors were significantly related to alcohol consumption during the pandemic. Subsequently, a high prosocial activity had been recorded to correlate with lower alcohol use and other antisocial behavior (48), and, vice versa, deviant peer associations were linked to higher rates of alcohol misuse (49).

Interestingly, the activation of the ventral striatum to reward stimuli from prosocial activities was predictive of lower risk-taking behaviors, including illicit substance use (50). More specifically, school attendances (51) and positive prosocial experiences were associated with reduced alcohol use (52), and parental warmth directed adolescents toward more prosocial peers (49). Prosocial attributes are known to correlate with better self-regulatory capacity (53) and could be focused on those who would form a higher belief in moral order (54), in turn mediating the reduction in odds of alcohol misuse. These could be valuable and applicable avenues to explore digitally to enhance prosocial affinity among adolescents and curb alcohol consumption. In light of the shifting psychiatric health provision in many countries (55), these linkages presented the necessity to maintain addiction services, particularly toward the subgroup of vulnerable adolescents.

### Cigarettes

Overall, the rate of current cigarette smoking during the COVID-19 pandemic, 3.1%, was lower compared to figures prior to the pandemic, 18.8% (56). This could be influenced by multiple factors affiliated to the pandemic; from reduced accessibility, availability, and increased perceived danger, the alternative results presented here could also be attributed to the concentrated education of detrimental correlation between COVID-19 and smoking (57). The disproportionate smoking tendencies between sexes had been recorded worldwide (58). Within the current sample, the male respondents were more likely to maintain cigarette smoking during the pandemic. This finding could be due to a higher rate of male smokers. Previous national surveys (59) and another study described that though higher perceived stress was seen among females, males reported a higher intensity of smoking and neuroendocrine reactivity (60). Secondly, as schools closed and learning shifted digitally, most adolescents were at home, which would present difficulty in continuing cigarette smoking in the presence of their parents. The majority of decreased smoking was reported by those in the high school, while those in the University reported increased smoking. This might have occurred as adolescents in the University could maintain living separately or having more freedom; supportively, older age was associated with higher odds of cigarette smoking during the pandemic. Interestingly, financial status did not present a clear pattern to changes in smoking habits, which could aggravate the economic burden amid the pandemic.

Neither living in lockdown provinces nor the proximity to COVID-19 cases were associated with smoking behavior, and nearly a fifth of the smoking respondents in PSBB provinces reported increased cigarette consumption. In Indonesia, many of the psychiatric and mental health resources were sidelined during the lockdown and pandemic. This should notify the stakeholders to maintain and even strengthen addiction health services in areas hard-hit by COVID-19, particularly toward adolescents. This study demonstrated that adolescents with high scores of CDS were associated with cigarette smoking during the pandemic; thus, identifying the at-risk adolescents and continuous cessation education and health support would be paramount.

### Drugs

In this study, less than half a percent of respondents consumed illicit drugs or abused prescriptions during the COVID-19 pandemic. This finding was in accordance with a survey from the Indonesian National Narcotics Board in 2018 that revealed a rate of only 0.44% of regular drug users among Indonesian adolescents (17). Studies assessing the impact of the COVID-19 pandemic on substance use disorder are still scarce in Indonesia. It was particularly notable that the reduced availability of and accessibility to drugs resulted in a stagnant, compared to decreased, use prevalence. A prior national survey (17) found that majority of Indonesian adolescents obtained substances by buying from their friends and just for experimental use, and the survey reported that the most accessible and available substance was cannabis and “tembakau gorilla” (a mixture of tobacco and synthetic cannabis). In the current study, cannabis and sedatives shared a similar proportion of usage. This finding resonated with the latest national survey that showed cannabis as one of the leading substances used by Indonesian adolescents, along with inhalants and analgesics (17). In this study, most substance users were female, unlike prior data, which noted the proportional prevalence of substance use in both sexes among junior high school students and male propensity among senior high school and University students. Concordantly, a prior Japanese post-disaster study highlighted that females were more inclined to resort to drug use (61). This could suggest a gendered proclivity of substance misuse under immense stressor. A study examining stress exposure and sex interaction revealed that greater responses were observed in the limbo-striatal and bilateral hippocampal regions for females than males (62), which could manifest as distinct stress-related complaints and impetus for substance use. This phenomenon could also be partially motivated by some biased views for female complaints resulting in higher sedative accessibility (63). However, the fact that a large proportion of the respondents in this study was female would bias this finding, and thus, further scrutiny is warranted.

The main strength of this study was being the first sizeable independent study on Indonesian adolescent substance use patterns and the first Indonesian study to analyze the patterns' changes during the COVID-19 pandemic. The responses gathered represented 33 out of 34 provinces in Indonesia, with response patterns resonating to real-world population density distribution. Some of the findings showed consistency with the national survey data, and relevant changes were recorded in this study.

However, there were some limitations in this study. First, some specifics of substance use attributes (e.g., history of use or disorder, detailed categories, procurement sources, and context of use) were not captured, thus requiring further research. Second, the study could not employ probability sampling or match to national census data due to the fast unraveling of the pandemic and limited available resources. Third, the digital and self-report surveys would pose response bias, e.g., social desirability and recall bias. Overall, the authors hoped that these findings provide preliminary insights for refining mental health and addiction policies and guidance for further research during and post-pandemic.

To conclude, the COVID-19 pandemic has given tons of impacts to humanity, including psychological well-being among adolescents. Our study showed that during the COVID-19 pandemic, rates of adolescent substance use in Indonesia were significant, with sizeable proportions reporting higher usage. In addition, our study showed a lower protective psychosocial attribute, i.e., prosocial behavior, during the lockdown. Therefore, early recognition of substance use is crucial, particularly during the COVID-19 pandemic. These findings also should urge the strengthening of adolescent addiction care during and after the pandemic.

## Data Availability Statement

The raw data supporting the conclusions of this article will be made available by the authors, without undue reservation.

## Ethics Statement

The studies involving human participants were reviewed and approved by Institutional Ethics Committee of the Faculty of Medicine, Universitas Indonesia–Dr. Cipto Mangunkusumo General Hospital. Written informed consent to participate in this study was provided by the participants' legal guardian/next of kin.

## Author Contributions

KS and EH designed and supervised the study. KS, EH, LTS, BM, and HC contributed data or analysis tools. KS, EH, LTS, BM, HC, AL, A, and LPS collected the data and wrote the initial manuscript. KS, EH, and LTS performed the data analysis. KS, EH, LTS and BM revised the manuscript. KS secured funding for the study. All authors contributed to the article and approved the submitted version.

## Funding

This study received funding from the Ministry of Research and Technology/National Research and Innovation Agency of Republic of Indonesia through the Konsorsium Riset dan Inovasi Untuk Percepatan Penanganan Corona Virus Disease 2019 (COVID-19) (Ref: 106/FI/PKS-KCOVID-19.F/VI/2020). The funders had no role in the design, data collection, analysis and interpretation of data, write-up, and/or publication of this study.

## Conflict of Interest

The authors declare that the research was conducted in the absence of any commercial or financial relationships that could be construed as a potential conflict of interest.

## Publisher's Note

All claims expressed in this article are solely those of the authors and do not necessarily represent those of their affiliated organizations, or those of the publisher, the editors and the reviewers. Any product that may be evaluated in this article, or claim that may be made by its manufacturer, is not guaranteed or endorsed by the publisher.
